# Supplementary material for: Analysis of 3800-year-old Yersinia pestis genomes suggests Bronze Age origin for bubonic plague
Source: Nat Commun. 2018 Jun 8;9:2234. doi: 10.1038/s41467-018-04550-9 (PMC5993720; doi:10.1038/s41467-018-04550-9)
Supplement: Supplementary file 1 — Supplementary Information [file 41467_2018_4550_MOESM1_ESM.pdf]

Supplementary Information

Title: Analysis of 3,800-year-old *Yersinia pestis* genomes suggests Bronze Age origin for bubonic plague

Spyrou et al.

Supplementary Figures: 1-10

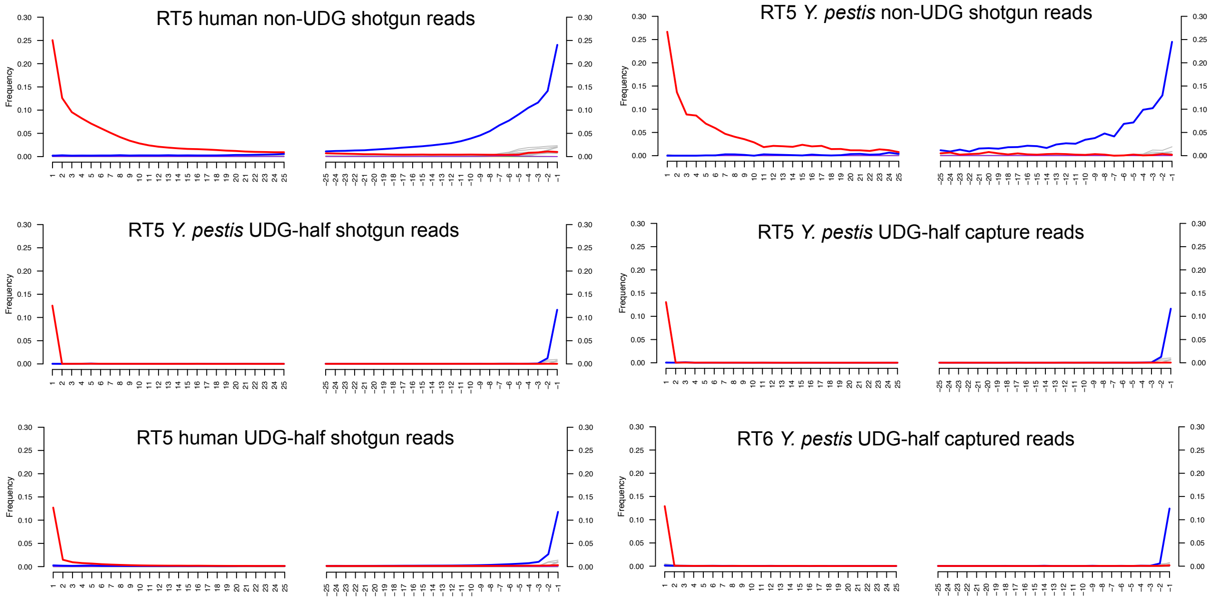

Supplementary Figure 1 – Ancient DNA damage profiles

Deamination profiles obtained from mapping of whole-genome shotgun sequenced and captured reads against the *Y. pestis* (CO92, chromosomal) and human (*hg19*, nuclear) reference genomes. Illumina double-stranded libraries were constructed using non-UDG[1] and partial UDG[2] protocols. Deamination profiles were produced using mapDamage2.0[3]. C > T changes at 5’ end of fragments are depicted in red, whereas G > A substitutions at 3’ end of fragments are depicted in blue.

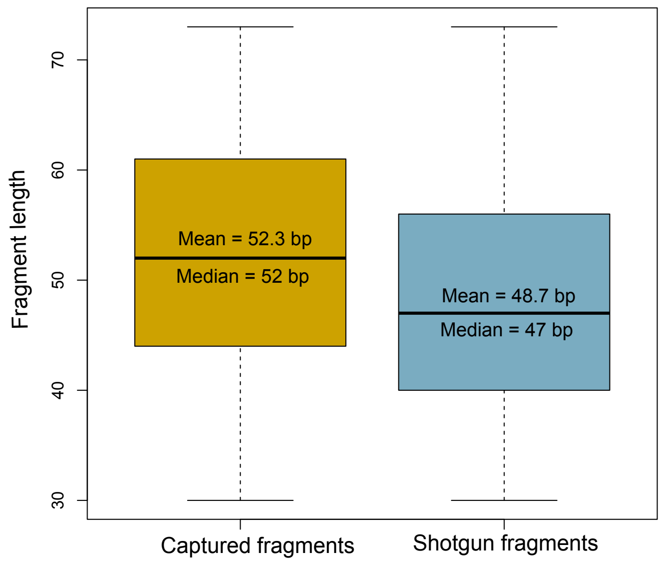

Supplementary Figure 2 – Shotgun sequenced vs captured fragment length distribution
Comparisons between RT5 *Y. pestis* fragment length distributions after deep shotgun sequencing, and after capture. Fragments used for this analysis were sequenced as single-ended 75 bp reads. A read length range of 30 - 74 bp was used for comparing the two datasets, where 77.7% and 89.4% of total mapped reads were retained from the capture and shotgun dataset, respectively. Comparison of these fractions revealed a 3.6 bp read length increase after capture (t-test, *P*-value < 2.2e-16). The plot was produced in R version 3.4.1[4].

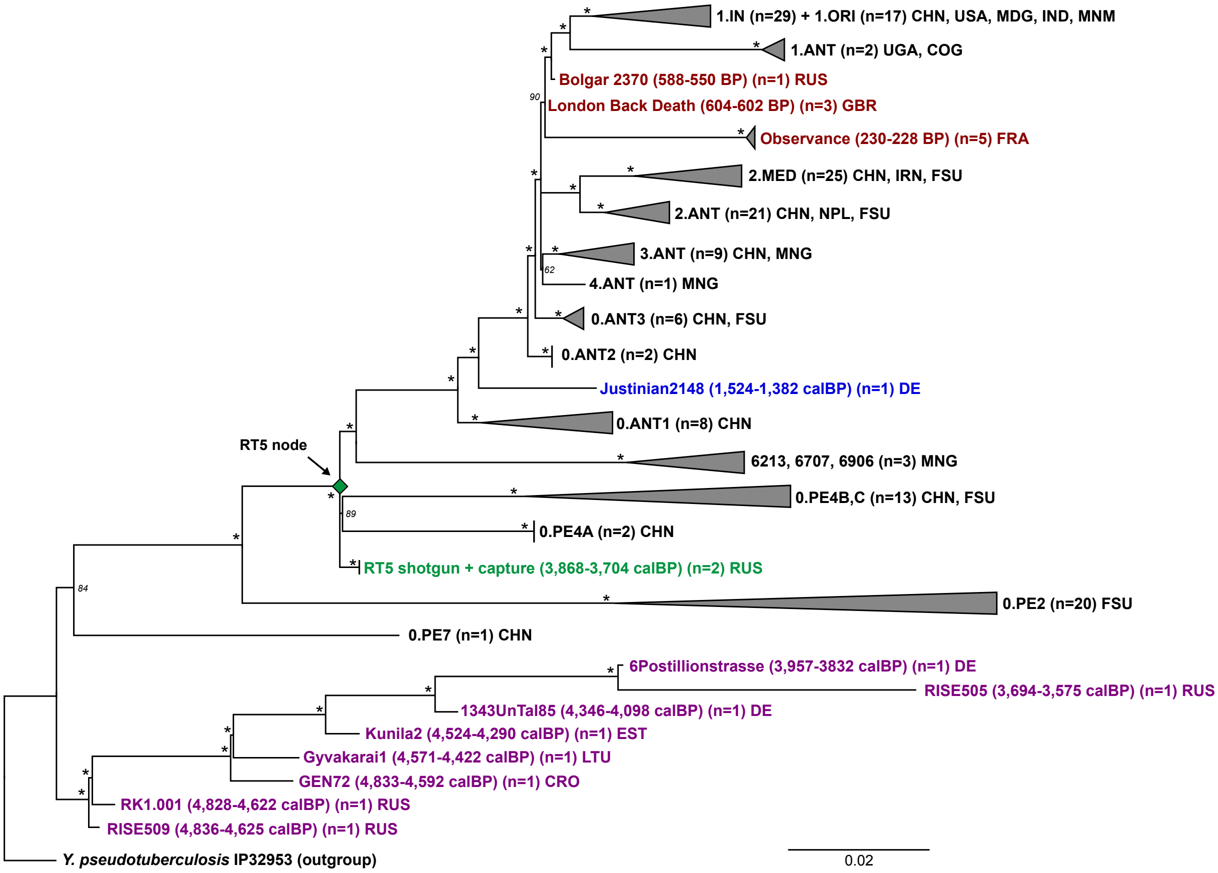

Supplementary Figure 3 – Maximum Likelihood phylogeny using all sites

The phylogenetic positioning of RT5 was assessed using a Maximum Likelihood approach and 1000 bootstrap iterations. The phylogenetic tree was produced using RaxML[5]. A worldwide dataset of *Y. pestis* chromosomal genomes (n=179) was used to reconstruct the phylogenetic tree, considering all variable positions (3,821 SNP positions). Main branches were collapsed to enhance the clarity of the phylogeny. The newly sequenced RT5 strain (green) was included in the phylogeny alongside eight Bronze Age strains belonging to the LNBA lineage (purple), a single Justinianic strain (blue), and nine second pandemic strains (red). Asterisks (*) denote bootstrap values > 95. The 2-sigma (95.4%) radiocarbon or archaeological dates of Bronze Age and historical strains are shown. Country or geographical region abbreviations are as follows: CHN (China), USA (United States of America), MDG (Madagascar), IND (India), IRN (Iran), MNM (Myanmar), RUS (Russia), GB (Great Britain), DE (Germany), FRA (France), MNG (Mongolia), NPL (Nepal), FSU (Former Soviet Union), CGO (Congo), and UGA (Uganda), LTU (Lithuania), EST (Estonia) and CRO (Croatia). See also Supplementary fig. 4 for the inferred phylogenetic positioning of RISE397.

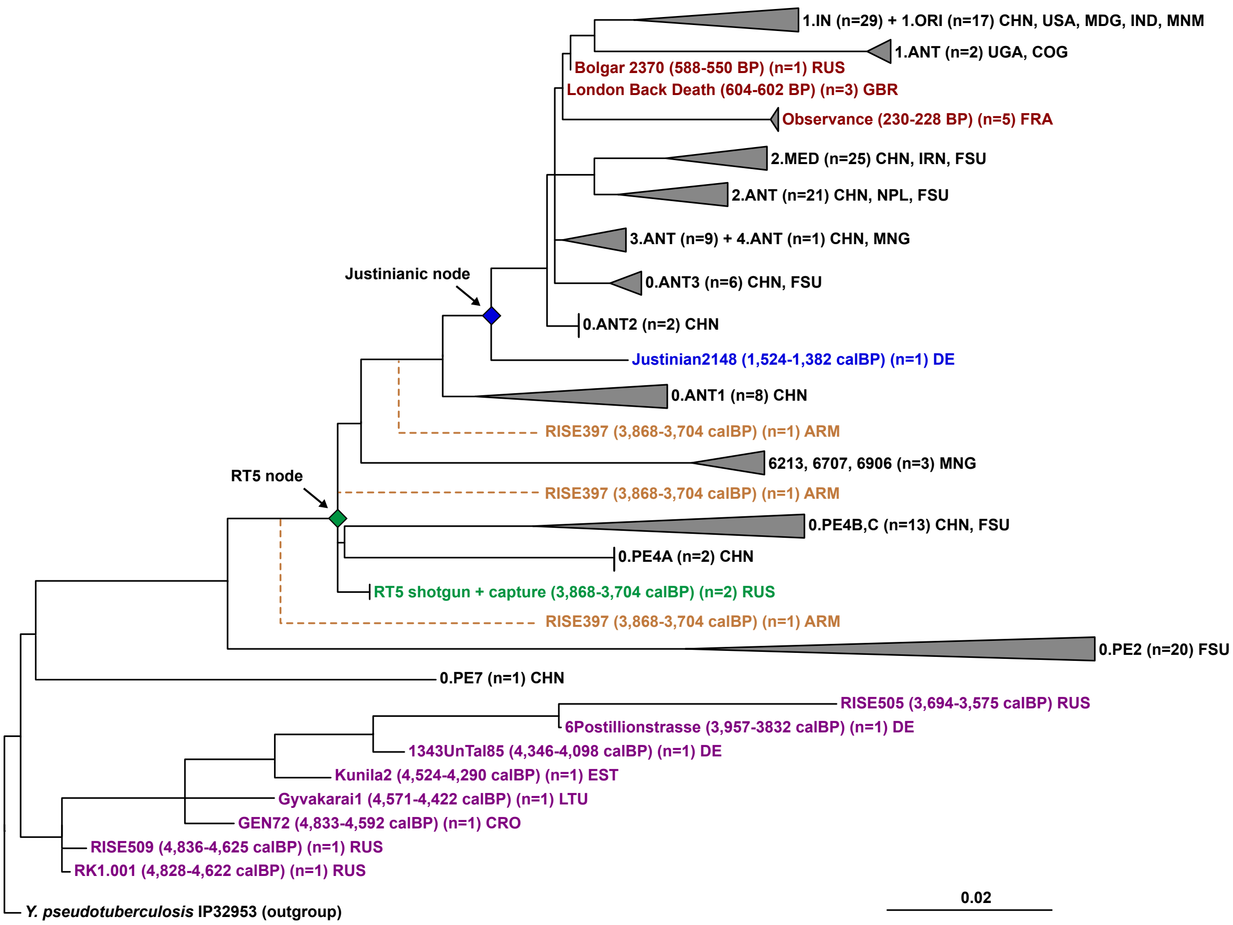

Supplementary Figure 4 – Inferred phylogenetic positioning of RISE397

The potential phylogenetic positioning of RISE397 was estimated by visual inspection of diagnostic SNPs and is illustrated here by dashed lines (see also Supplementary Data 3, 4) on the inferred Maximum Likelihood phylogeny (see Figure 2).

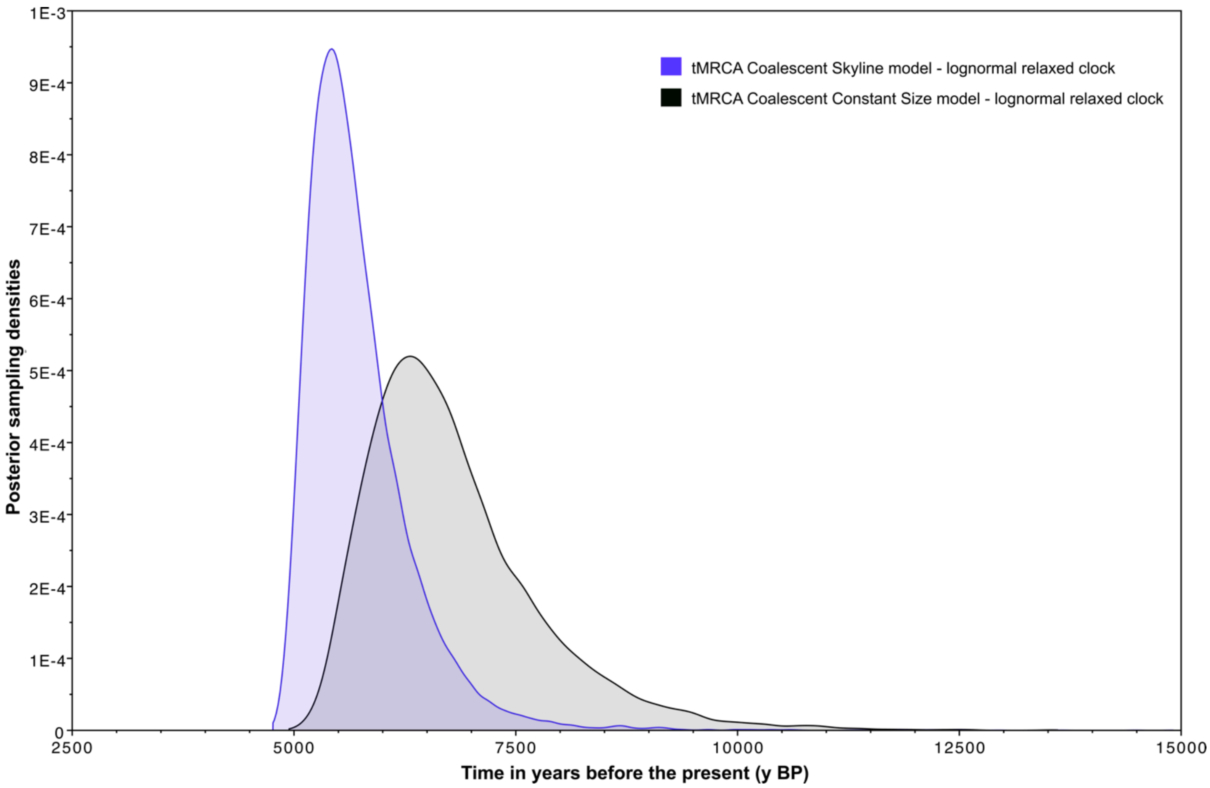

Supplementary Figure 5 – Posterior distributions of tMRCAs using Tracer v1.6
Overlapping posterior distributions of the time to the most recent common ancestor (tMRCA) for all *Y. pestis* using the Coalescent Constant Size[6] and Coalescent Bayesian Skyline[7] models in BEAST v1.8[8].

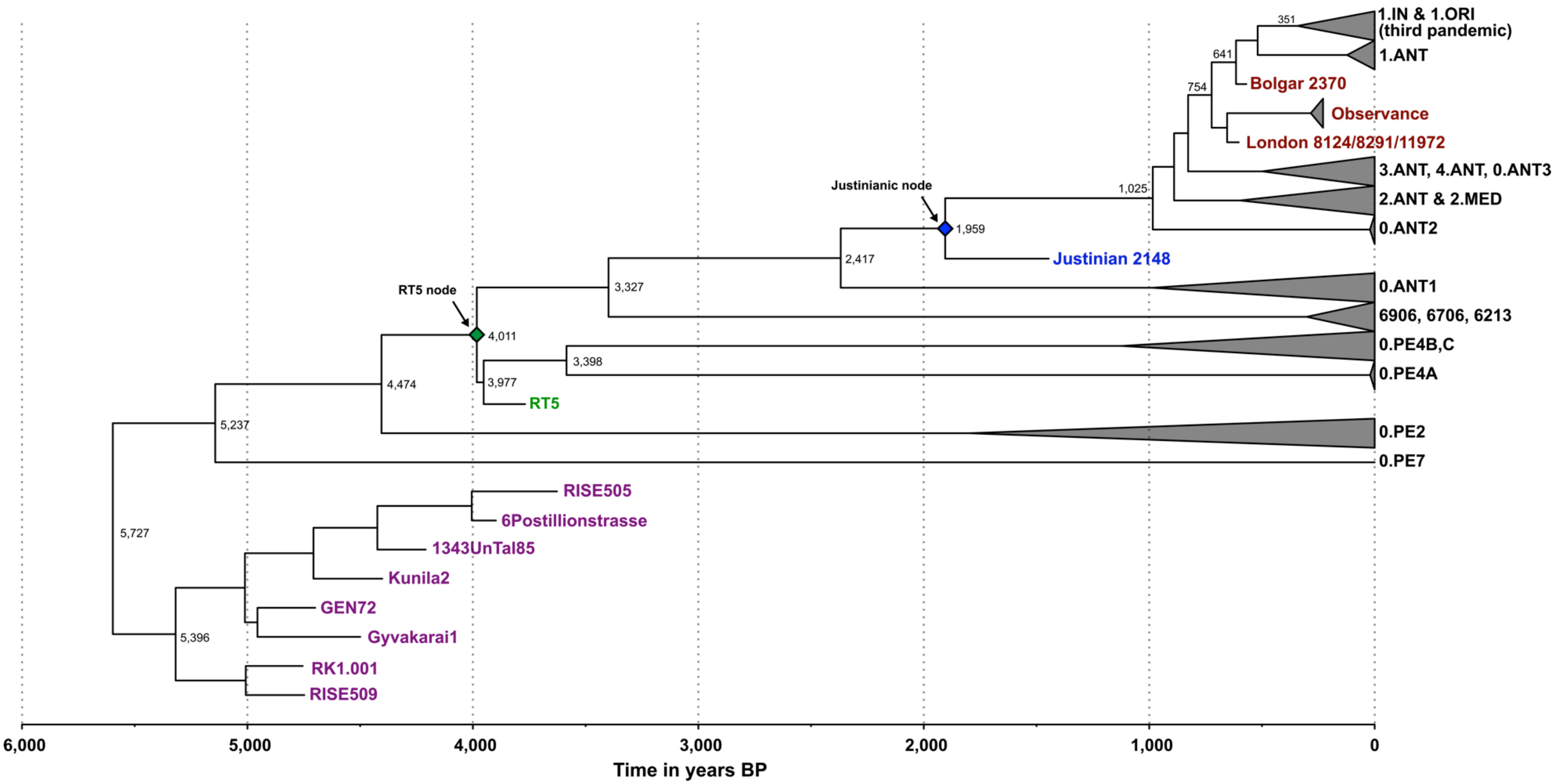

Supplementary Figure 6 - Maximum Clade Credibility tree

The MCC tree was produced using TreeAnnotator of BEAST v1.8[8] and is a product of demographic analysis based on the Coalescent Skyline model, summarizing 27,001 trees. The tree was visualized in FigTree v1.4.2 (http://tree.bio.ed.ac.uk/software/figtree/). It is presented in a temporal scale between 6,000 and 0 yBP, and the mean divergence dates of major *Y. pestis* lineages are indicated on each corresponding node.

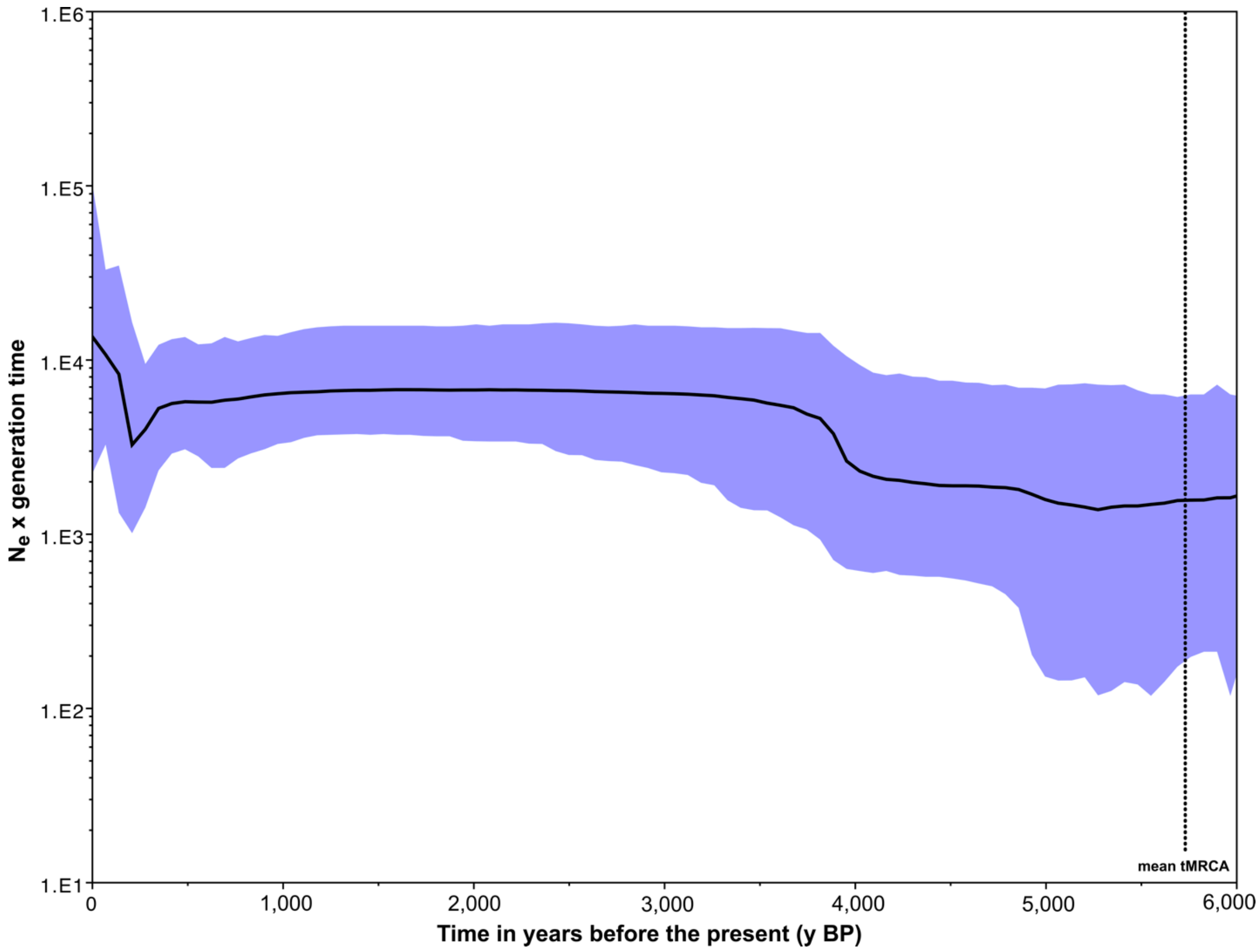

Supplementary Figure 7 – Coalescent Skyline plot

Depiction of effective population size (N_e) changes over time, as estimated by the Bayesian Coalescent Skyline model, implemented in BEAST v1.8[8]. The plot was produced in Tracer v1.6 (http://tree.bio.ed.ac.uk/software/tracer/). *Y. pestis* strains used for this analysis, overlap with ones used for the phylogenetic analysis (see Figure 2), excluding the outgroup (see Methods). The mean divergence date of all *Y. pestis* strains (5,727y BP) is shown with a dotted line.

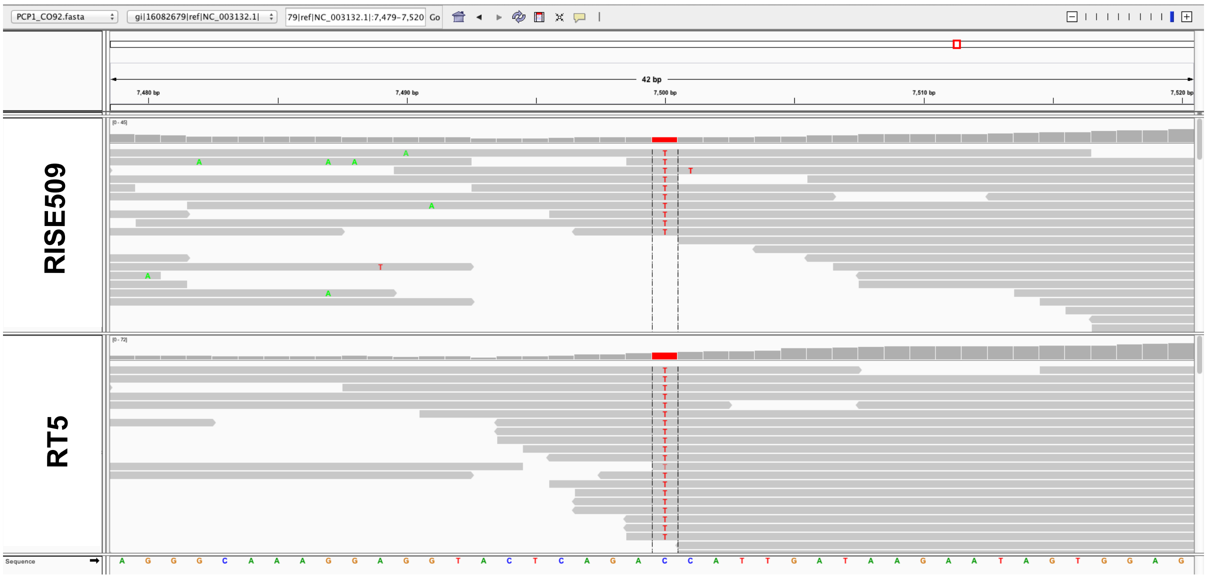

Supplementary Figure 8 – Pla genotype

RT5 and RISE509 *pla* genotype (pPCP1 plasmid), shown using an IGV[9] screenshot. The ancestral 'T' variant at position 7,500 on pPCP1 (CO92) confers an isoleucine at amino acid position 259 in *pla*[10].

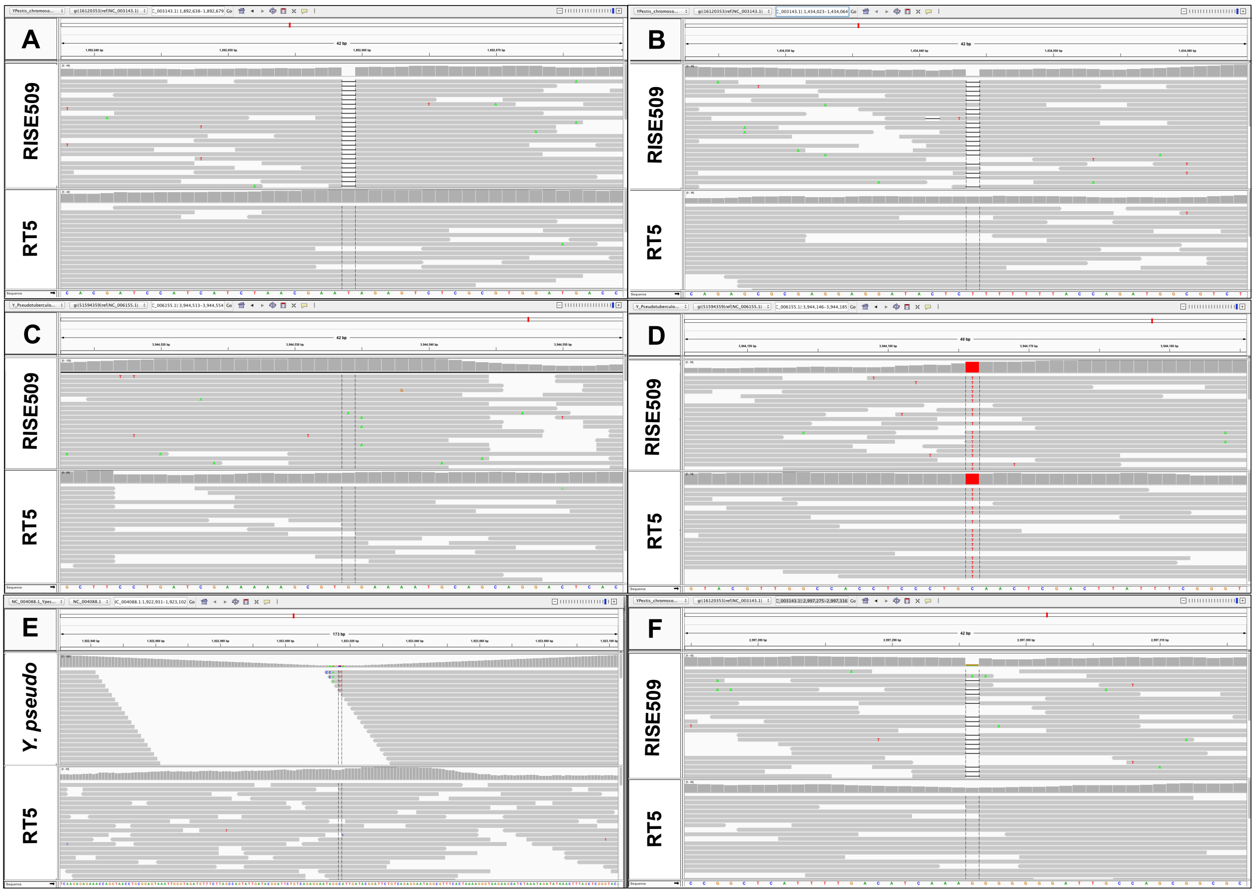

Supplementary Figure 9 – Visual inspection of virulence factors associated with *Y.pestis* immune evasion and flea adaptation. IGV[9] screenshots of virulence genes affected by substitutions, single nucleotide InDels or within gene duplications. RT5 was compared to either RISE509 or *Y. pseudotuberculosis* IP32953 for the following genes: (A) *flhD*, (B) PDE-2, (C) PDE-3, (D) PDE-3-*pe'*, (E) *rcsA*, (F) *ureD*. See main Methods section for additional information on the affected regions.

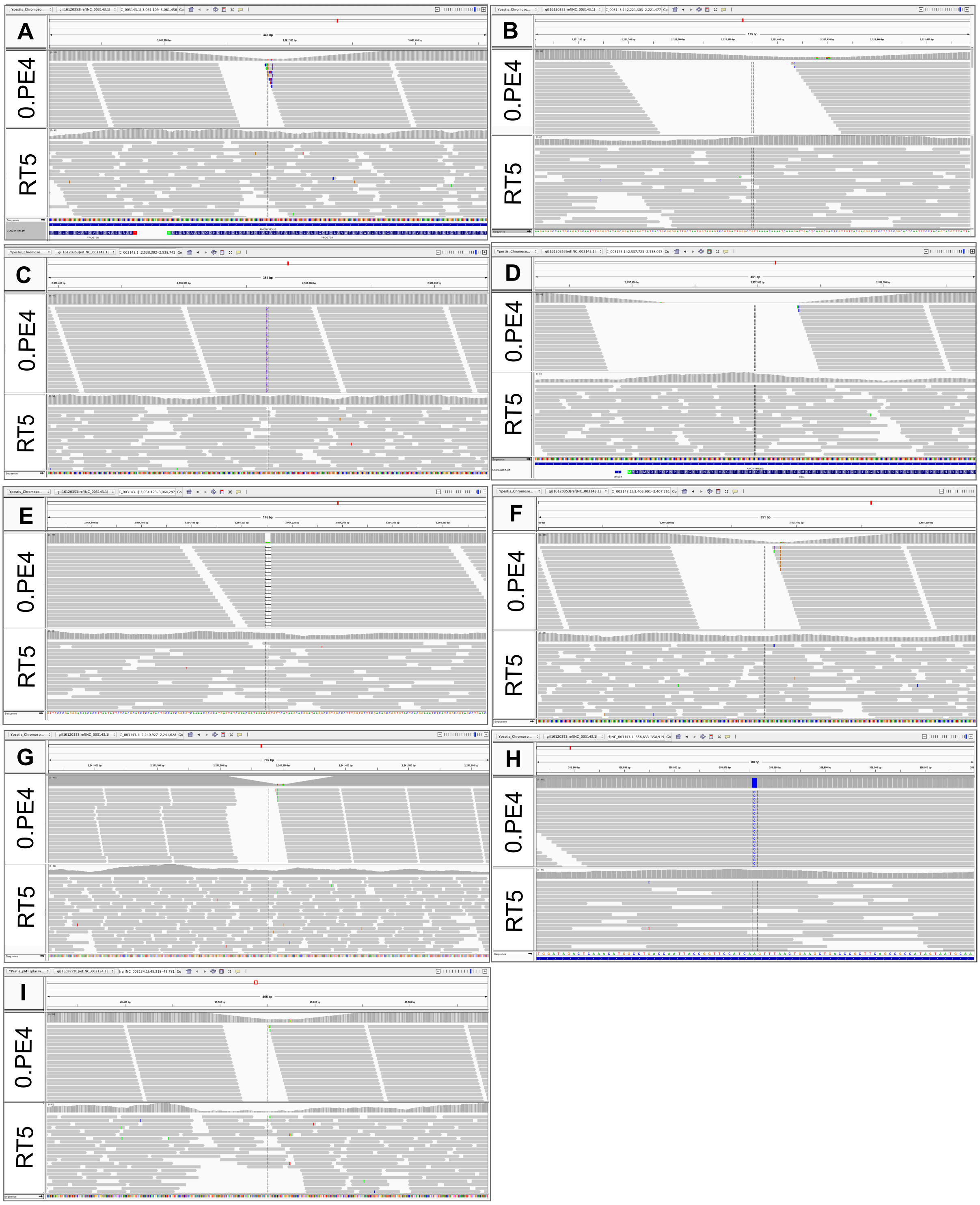

Supplementary Figure 10 – Visual inspection of 0.PE4-affected genes in RT5

The depicted genetic loci have been suggested to contribute to a decreased virulence in 0.PE4 Microtus 91001. Here we show IGV[9] screenshots of the RT5 genotype compared to that of 0.PE4 Microtus 91001 strain. The genes shown here are the ones either disrupted by IS elements, affected by small deletions, or by substitutions, in 0.PE4 Microtus 91001: (A) YPO2729 (B) YPO1956, (C) YPO2258 (G insertion) (D) YPO2258 (or *araC*) (E) YPO2731 (F) YPO3049 (or *hutC*), (G) YPO1973 (H) YPO0348 (I) YPMT1.43c. Refer to Supplementary Table 10 and Figure 3 for a description of these and other affected regions.

Supplementary Tables: 1-11

Supplementary Table 1 - *Yersinia pestis* screening statistics for all non-UDG Samara libraries. Specimens considered as putatively positive are highlighted.

Sample	Raw Reads	Pre-processed Reads	Unique Mapping Reads (CO92)	Endogenous DNA (%)	Reads assigned by MALT to *Y. pestis/Y. pseudotuberculosis* complex
RT1	7,070,200	6,862,786	10	0.000	0/0
RT2	7,760,547	7,477,994	42	0.001	0/0
RT3	6,541,880	6,229,799	38	0.001	0/37
RT4	6,255,907	6,043,051	10	0.000	0/0
RT5	7,258,101	7,003,670	7,186	0.117	1,423/7,627
RT6	7,017,656	6,706,316	82	0.001	0/71
RT7	7,928,892	7,638,467	26	0.000	0/0
RT8	5,969,436	5,766,028	34	0.001	0/35
RT9	8,215,620	7,957,649	15	0.000	0/0

Supplementary Table 2 - Human DNA screening of all non-UDG Samara libraries after mapping against *hg19*. The specimen that was pursued for further analysis is highlighted.

Sample	Raw Reads	Pre-Processed Reads	Unique Mapped Reads	Endogenous DNA (%)	Duplication Factor	Mean Fragment Length	GC content (%)
RT1	7,070,200	6,862,786	2,128,479	33.5	1.08	62.1	45.8%
RT2	7,760,547	7,477,994	17,842	0.3	1.07	60.1	43.6%
RT3	6,541,880	6,229,799	10,065	0.2	1.08	52.7	42.7%
RT4	6,255,907	6,043,051	6,687	0.1	1.07	61.7	44.9%
RT5	7,258,101	7,003,670	2,008,157	31.3	1.09	53.5	46.2%
RT6	7,017,656	6,706,316	77,968	1.2	1.07	60.5	43.0%
RT7	7,928,892	7,638,467	13,526	0.2	1.10	61.8	43.5%
RT8	5,969,436	5,766,028	17,314	0.3	1.08	52.3	46.4%
RT9	8,215,620	7,957,649	5,432	0.1	1.08	64.0	44.2%

Supplementary Table 3 - Genome reconstruction statistics after capture and deep shotgun sequencing of partially UDG-treated libraries.

Data type	Sample	Reference used for mapping	Unique Mapping Reads	Endogenous DNA (%)	Duplication Factor	Mean Coverage	Coverage across reference >= 3X	GC content (%)
Shotgun Reads	RT5	CO92 Chrom.	826,585	0.094	1.21	9.2	89.02%	48.61
		CO92 pCD1	27,688	0.005	1.79	21.4	93.45%	46.89
		CO92 pMT1	21,991	0.003	1.56	11.8	92.34%	50.85
		CO92 pPCP1	9,228	0.003	3.17	57.0	87.41%	45.85
		Human HG19	251,410,787	27.40	1.22	4.2	54.93%	47.62
Captured Reads	RT5	CO92 Chrom.	2,566,811	33.85	2.07	32.3	93.52%	48.38
		CO92 pCD1	64,303	1.738	4.23	57.6	95.03%	46.50
		CO92 pMT1	61,412	1.671	2.69	37.4	95.70%	50.50
		CO92 pPCP1	11,000	0.784	11.15	80.3	87.25%	46.21
	RT6	CO92 Chrom.	191,079	7.161	7.48	1.9	28.58%	48.82
		CO92 pCD1	7,467	0.380	10.15	5.0	55.40%	48.12
		CO92 pMT1	6,554	0.264	8.04	3.2	46.00%	50.76
		CO92 pPCP1	2,938	0.175	13.41	14.9	67.98%	49.00

Supplementary Table 4 – Results of X-chromosomal contamination estimates for RT5.

Methods	Version	MoM	SE(MoM)	ML	SE(ML)
Method1	old_llh	0.005003	6.786513e-04	0.005958	3.950846e-13
Method1	new_llh	0.004986	6.808932e-04	0.005957	2.329628e-13
Method2	old_llh	0.004085	1.209904e-03	0.004209	4.900471e-14
Method2	new_llh	0.004071	1.213368e-03	0.004206	2.082066e-13

Supplementary Table 5 - Y-chromosomal SNP positions present in RT5.

Haplogroup	SNP name	Other names	rsID	GRCh37	Ancestral	Derived	RT5	Coverage
R1a	L62	M513; PF6200	rs17222573	17,891,241	A	G	G	2
R1a	L63	M511; PF6203	rs17307677	18,162,834	T	C	C	1
R1a	L146	M420; PF6229	rs17250535	23,473,201	T	A	A	4
R1a1	Page65.2	PF6234; SRY1532.2; SRY10831.2	rs2534636	2,657,176	C	T	T	2
R1a1	M459	PF6235	rs17316227	6,906,074	A	G	G	1
R1a1	L120	M516; PF6236	rs17307105	15,879,017	A	G	G	1
R1a1a	M515	NA	rs17221601	14,054,623	T	A	A	2
R1a1a	L168	NA		16,202,177	A	G	G	3
R1a1a1	M417	NA	rs17316771	8,533,735	G	A	A	3
R1a1a1	Page7	NA	rs34297606	14,498,990	C	T	T	4
R1a1a1b	S441	Z647	rs112284571	7,683,058	G	A	A	2
R1a1a1b	S224	Z645	rs111731595	8,245,045	C	T	T	3
R1a1a1b2	F992	S202; Z93		7,552,356	G	A	A	1

Supplementary Table 6 - Radiocarbon dating results of two individuals from the Mikhailovsky II burial site.

Individual	Material	Labno. MAMS	^{14}C age [yrs BP]	Cal 2-sigma (95.4%)
RT5	Tooth	29430	3,517 ± 27	3,868-3,704 calBP
RT6	Tooth	29431	3,499 ± 25	3,842-3,696 calBP

Supplementary Table 7 - Unique *Y. pestis* SNP positions identified in individual RT5, and their respective genotype in ancient genomes included in this study.

**Position**	**Gene /Locus**	**aa* change**	**Reference (CO92)**	**RT5**	**RT6**	**GEN72**	**RK1.001**	**Kunila2**	**Gyvakarai1**	**RISE505**	**RISE509**	**6Post**	**1343UnTal85**	**Justinian 2148**	**Black Death 8124/8291/11972**
1,339,231	YPO1189	N/A	C	A	A	.	.	.	.	.	.	.	.	.	.
2,961,749	*chb*	H > T	C	T	N	.	.	.	.	.	.	.	.	.	.
3,369,465	*cysP*	F > L	G	A	A	.	.	.	.	.	.	.	.	.	.
3,620,361	*hmwA*	G > R	C	A	A	.	.	N	.	.	.	N	.	.	.
4,182,150	*purH*	A > S	G	T	T	.	.	.	.	.	.	.	.	.	.

*Amino acid

Supplementary Table 8 – Demographic model comparisons using PS/SS sampling[11] in BEASTv1.8[8]. The table shows the marginal likelihood estimates (MLE) produced for the two models by both analyses.

	Coalescent Constant Size	Coalescent Bayesian Skyline
**Path sampling MLE**	-9535.76	-9,526.68
**Stepping stone sampling MLE**	-9535.46	-9,528.37

Supplementary Table 9 - *Y. pestis* dating result comparisons between Coalescent Constant Size and Coalescent Skyline demographic models using BEASTv1.8[8]

Lineage divergence	**Coalescent Constant Size[6]** 95% HPD (mean y BP)	**Coalescent Bayesian Skyline[7]** 95% HPD (mean y BP)
**MRCA***	6,797 (5,299-8,743)	5,727 (4,909-6,842)
**0.PE7**	6,225 (4,569-8,076)	5,237 (4,248-6,346)
**0.PE2**	4,879 (4,003-6,009)	4,474 (3,936-5,158)
**RT5**	4,089 (3,761-4,513)	4,011 (3,760-4,325)
**Justinianic**	1,925 (1,501-2,440)	1,959 (1,500-2,536)
**Black Death**	780 (604-1,029)	754 (603-989)

*MRCA here refers to the most recent common ancestor of all *Y. pestis* isolates, i.e. the root of the tree.

Supplementary Table 10 - List of regions/loci affected in 0.PE4 Microtus 91001 and considered as contributors to its decreased virulence.

Locus name in CO92	Position Start	Position End	Locus name in Mictorus 91001	Description[12,13]
YPO0348	358,526	359,962	*aspA*	C to A substitution at position 358,876 (CO92)[*]
YPO1956	2,221,367	2,221,645	YP1700	IS*285* insertion (disrupted)[*]
YPO1973	2,240,953	2,241,720	YP1715	IS*100* insertion (disrupted)[*]
YPO2258 (*araC*)	2,537,797	2,538,729	YP2054	112 bp deletion 26 bp downstream of start codon + G insertion 775 bp downstream of start codon[*]
YPO2729	3,061,203	3,061,550	YP2435	IS*285* insertion (disrupted)[*]
YPO2731	3,062,324	3,064,363	YP2433	2 bp deletion 153 bp downstream of start codon[*]
YPO3049	3,406,449	3,408,218	YP2671	7 bp deletion 1,125 bp downstream of start codon[*]
YPO1986-1987	2,254,897	2,257,189	N/A	Disrupted/Absent[**]
YPO2096-2135	2,370,557	2,403,216	N/A	Disrupted/Absent[**]
YPO2469	2,769,322	2,769,654	N/A	Disrupted/Absent[**]
YPO2487-2489	2,788,665	2,790,115	N/A	Disrupted/Absent[**]
YPO3046-3047	3,402,002	3,404,908	N/A	Disrupted/Absent[**]
YPMT1.43c (pMT1)	4,5098	45,742	pMT056	20 bp deletion 69 bp downstream of start codon[*]

[*]See Supplementary Figure 10 for visual inspection of all InDels and mutations in 0.PE4
[**]See Figure 3 of main text for loci absent in 0.PE4

Supplementary Table 11 - Newly identified SNPs that were excluded from comparative variant calling analysis in addition to previously defined regions.

Position in CO92	Reference	Variant	Description	Strains identified in
1,029,500	A	G	SNP*	Georgia 1413 (Zhgenti *et al.*, 2015[14])
1,029,502	A	C	SNP*	Georgia 1413 (Zhgenti *et al.*, 2015[14])
1,029,503	A	C	SNP*	Georgia 1413 (Zhgenti *et al.*, 2015[14])
1,361,705	T	C	SNP*	6304 (Kislichkina *et al.*,2015[15])
1,361,707	A	G	SNP*	6304 (Kislichkina *et al.*,2015[15])
1,361,719	G	T	SNP*	6304 (Kislichkina *et al.*,2015[15])
1,687,299	G	T	SNP*	6904 (Kislichkina *et al.*,2015[15])
1,687,300	T	C	SNP*	6904 (Kislichkina *et al.*,2015[15])
1,687,301	T	C	SNP*	6904 (Kislichkina *et al.*,2015[15])
3,489,416	G	A	SNP*	1.ANT1_UG05-0454**
3,489,419	G	A	SNP*	1.ANT1_UG05-0454**
3,489,428	G	T	SNP*	1.ANT1_UG05-0454**
3,489,429	A	T	SNP*	1.ANT1_UG05-0454**
3,860,629	A	G	SNP*	0.PE7b_620024[16]
3,860,637	A	C	SNP*	0.PE7b_620024[16]
3,860,639	A	T	SNP*	0.PE7b_620024[16]
4,273,931	C	A	SNP*	0.ANT3a_CMCC38001[16]
4,273,933	C	T	SNP*	0.ANT3a_CMCC38001[16]
4,273,941	A	T	SNP*	0.ANT3a_CMCC38001[16]
4,273,942	A	T	SNP*	0.ANT3a_CMCC38001[16]
4,355,693	C	A	SNP*	6216 (Kislichkina *et al.*,2015[15])
4,355,759	T	C	SNP*	6216 (Kislichkina *et al.*,2015[15])
4,355,760	A	G	SNP*	6216 (Kislichkina *et al.*,2015[15])
3,939,869	T	A	SNP*	6304 (Kislichkina *et al.*,2015[15])
3,939,870	T	C	SNP*	6304 (Kislichkina *et al.*,2015[15])
3,939,872	T	C	SNP*	6304 (Kislichkina *et al.*,2015[15])
358,876	A	C	homoplastic SNP	shared between LNBA, 0.PE4 and 2.MED3i
1,805,037	C	A	homoplastic SNP	shared between 0.PE2 and 0.PE4

*SNPs that appear within potentially recombining regions as defined by ClonalFrameML[17]
**NCBI Accession NZ_AAYR00000000 (See Supplementary Data 2)

Supplementary Methods

Archaeological context and sample information

Mikhailovsky II burial site, Samara, Russia

Between the end of the Middle Bronze Age (MBA) and the beginning of the Late Bronze Age (LBA) there is a cultural expansion observed in the Eurasian steppes that results in a series of genetically and culturally related populations extending from the Altai all the way to Europe during the LBA[18-20]. The two most widespread LBA cultures are the 'Andronovo' and 'Srubnaya' and together their subsistence economy is often described as 'complex agro-pastoralism'[21]. While the 'Andronovo' is found to occupy most of the area east of the Ural Mountains extending from the southern Urals to the Altai, the 'Srubnaya' or 'Timber grave' culture was dominant in the European steppes, west of the Ural Mountains. Here, we analyse material from the Mikhailovsky II burial site, which was excavated in 2015 and is one of numerous kurgan cemeteries identified in the Samara Oblast. It consists of seven kurgan burials, and is chronologically associated to the 'Pokrovka' phase (3,900-3,750 BP) of the 'Srubnaya' culture (3,850-3,150 BP) (radiocarbon dates produced in this study provided in Supplementary Table 6), also referred to as the 'proto-Srubnaya' that is considered the earliest phase of the LBA in the Samara Oblast. All sex and age groups were represented in this cemetery. We analysed nine individuals buried in three kurgans and identified two individuals buried in the same kurgan (see Supplementary Figure 1) to be positive for *Y. pestis.* According to anthropological analysis these were a 30-40 year-old male (RT5) and 35-45 year-old female (RT6).

Read-processing, human DNA screening and genome reconstruction

The sequenced, non-UDG, double-stranded libraries, prepared for screening from 10 µl of extract, were pre-processed using EAGER v1.92[22], for removing adaptors, length filtering (kept all reads of length > 30 bp), and quality filtering (q 20). To assess the amount of endogenous DNA in each dataset, reads where then mapped with BWA[23], which is implemented in EAGER, against the human *hg19* genome, using a seedlength of 32, –n 0.01, and a minimum mapping quality of 0.

The deep shotgun-sequenced, partial-UDG treated[2], RT5 library was pre-processed for genome reconstruction in the following way: Adaptors were removed using leeHom[24], and reads were then used as input in EAGER for subsequent length and quality filtering, as well as BWA mapping as mentioned above. Finally, 2 bp were clipped from both the 3' and 5'

ends of reads after mapping, to avoid the interfering of damaged bases with subsequent SNP calling.

RT5 genetic sex determination & contamination estimation

We performed sex identification of the deep-sequenced RT5 using two well-tested methods called Rx and Ry previously described in Skoglund et al[25] and Mittnik et al[26]. We calculated the number of reads mapping against the X and Y chromosome and compared the rates to the total number of reads mapping against the autosomes. Based on this ratio, we then decided upon a threshold to assess whether our specimen could be identified as male or female. Individual RT5 showed 4,476,135 reads mapping to the X chromosome and 382,860 reads mapping onto the Y chromosome. The Rx and Ry are calculated to be 0.449 and 0.079 respectively, which are consistent RT5 being a male.

We performed a X-chromosomal contamination test following an approach introduced by Rasmussen et al[27] and implemented in the ANGSD software suite[28]. We found a total number of 21,425 SNPs on chromosome X covered at least twice and applied the "MoM" and "ML" estimate from "Method 1" and "Method 2" likelihood computation. We determined a consistent contamination estimate of about 0.5% from different methods (Supplementary table 4).

RT5 Y-chromosomal haplogroup identification

We were able to determine the Y chromosomal haplogroup by examining a set of diagnostic positions on chromosome Y using the ISOGG database (http://isogg.org/). For this, we restricted our analysis to only include reads with a mapping quality higher than 30. We determined haplogroups by identifying the most derived Y chromosomal SNP in individual RT5. The sample has a derived allele at R1a1a1b2-F992: G→A, however only with coverage of 1-fold. As the low coverage at that position might cause a misidentification of the haplogroup, our individual was not assigned to haplogroup R1a1a1b2. We were instead able to find multiple upstream mutations assigning our individual to R1a1a1b and R1a1a1, suggesting that placement of individual RT5 in Y chromosomal haplogroup R1a1a1b is ultimately correct.

RT5 mitochondrial haplogroup analysis

We used EAGER v1.92[29] to reconstruct the RT5 mitochondrial genome and created a quality filtered (q=30) consensus sequence using the tool schmutzi[30]. Using MitoTool[31], we determined the maternal haplogroup of this individual to be U2e2a with the following variants:

73, 152, 217, 263, 508, 750, 1438, 1719, 1811, 2706, 3106A, 3720, 3849, 4553, 4736A, 4746d, 4749, 4750G, 4754-4771d, 5390, 5426, 6045, 6152, 7028, 7058, 8473, 10876, 11467, 11719, 12308, 12372, 12557, 13020, 13734, 14766, 15326, 15891, 15907, 16051, 16092, 16129C, 16183C, 16189, 16362, 16519.

1240K pulldown

We used a dataset of 1240K SNPs and pulled down variants for these alleles as previously published in Lazaridis et al.[32] and obtained a mean depth of 4.2-fold and a total coverage of 1,100,070 SNPs. On this dataset, we performed several population genetic analyses to investigate the genetic ancestry of RT5 and its relationship to modern and ancient populations from West Eurasia[32-35].

Principal Component Analysis

We used smartpca (version: 13050)[36], part of the EIGENSOFT package[37,38], to carry out Principal Component Analysis (PCA). We performed PCA on present-day West Eurasian populations and then projected the ancient samples using the lsqproject: YES option, which accounts for samples with substantial missing data. We did not perform any outlier removal iterations (numoutlieriter: 0). We set all other options to default.

ADMIXTURE analysis

We performed an ADMIXTURE[39] analysis after pruning the data for linkage disequilibrium in PLINK[40] with the parameters --indep-pairwise 200 25 0.4 retaining 298,692 SNPs for the Human Origin dataset. We ran ADMIXTURE with default 5-fold cross validation, varying the number of ancestral populations between $K = 2$ and $K = 16$ in bootstraps of 100 with different random seeds. We used 331 ancient samples as well as 2583 present-day individuals from worldwide populations for our analysis. The lowest cross-validation errors could be observed with $K=12$.

Supplementary References

1 Meyer, M. & Kircher, M. Illumina sequencing library preparation for highly multiplexed target capture and sequencing. *Cold Spring Harb Protoc* **2010**, pdb prot5448, doi:10.1101/pdb.prot5448 (2010).

2 Rohland, N., Harney, E., Mallick, S., Nordenfelt, S. & Reich, D. Partial uracil-DNA-glycosylase treatment for screening of ancient DNA. *Philos Trans R Soc Lond B Biol Sci* **370**, 20130624, doi:10.1098/rstb.2013.0624 (2015).

3 Jonsson, H., Ginolhac, A., Schubert, M., Johnson, P. L. & Orlando, L. mapDamage2.0: fast approximate Bayesian estimates of ancient DNA damage parameters. *Bioinformatics* **29**, 1682-1684, doi:10.1093/bioinformatics/btt193 (2013).

4 R Core Team. R: A language and environment for statistical computing. *R Foundation for Statistical Computing, Vienna, Austria* (2015).

5 Stamatakis, A. RAxML version 8: a tool for phylogenetic analysis and post-analysis of large phylogenies. *Bioinformatics* **30**, 1312-1313, doi:10.1093/bioinformatics/btu033 (2014).

6 Kingman, J. F. C. The coalescent. *Stoch Process Their Appl* **13**, 235-248 (1982).

7 Drummond, A. J., Rambaut, A., Shapiro, B. & Pybus, O. G. Bayesian coalescent inference of past population dynamics from molecular sequences. *Mol. Biol. Evol.* **22**, 1185-1192, doi:10.1093/molbev/msi103 (2005).

8 Drummond, A. J. & Rambaut, A. BEAST: Bayesian evolutionary analysis by sampling trees. *BMC Evol. Biol.* **7**, 214 (2007).

9 Thorvaldsdóttir, H., Robinson, J. T. & Mesirov, J. P. Integrative Genomics Viewer (IGV): high-performance genomics data visualization and exploration. *Brief. Bioinformatics* **14**, 178-192 (2013).

10 Zimbler, D. L., Schroeder, J. A., Eddy, J. L. & Lathem, W. W. Early emergence of Yersinia pestis as a severe respiratory pathogen. *Nat. Commun.* **6**, 7487 (2015).

11 Baele, G., Lemey, P. & Vansteelandt, S. Make the most of your samples: Bayes factor estimators for high-dimensional models of sequence evolution. *BMC Bioinformatics* **14**, 85, doi:10.1186/1471-2105-14-85 (2013).

12 Zhou, D. *et al.* Genetics of metabolic variations between Yersinia pestis biovars and the proposal of a new biovar, microtus. *J. Bacteriol.* **186**, 5147-5152 (2004).

13 Zhou, D. & Yang, R. Molecular Darwinian evolution of virulence in Yersinia pestis. *Infect. Immun.* **77**, 2242-2250, doi:10.1128/IAI.01477-08 (2009).

14 Zhgenti, E. *et al.* Genome Assemblies for 11 Yersinia pestis Strains Isolated in the Caucasus Region. *Genome Announc* **3**, e01030-01015, doi:10.1128/genomeA.01030-15 (2015).

15 Kislichkina, A. A. *et al.* Nineteen Whole-Genome Assemblies of Yersinia pestis subsp. microtus, Including Representatives of Biovars caucasica, talassica, hissarica, altaica, xilingolensis, and ulegeica. *Genome Announc* **3**, e01342-01315, doi:10.1128/genomeA.01342-15 (2015).

16 Cui, Y. *et al.* Historical variations in mutation rate in an epidemic pathogen, Yersinia pestis. *Proc Natl Acad Sci U S A* **110**, 577-582, doi:10.1073/pnas.1205750110 (2013).

17 Didelot, X. & Wilson, D. J. ClonalFrameML: efficient inference of recombination in whole bacterial genomes. *PLoS Comp. Biol.* **11**, e1004041 (2015).

18 Haak, W. *et al.* Massive migration from the steppe was a source for Indo-European languages in Europe. *Nature* **522**, 207-211, doi:10.1038/nature14317 (2015).

19 Allentoft, M. E. *et al.* Population genomics of Bronze Age Eurasia. *Nature* **522**, 167-172, doi:10.1038/nature14507 (2015).

20 Mathieson, I. *et al.* Genome-wide patterns of selection in 230 ancient Eurasians. *Nature* **528**, 499-503, doi:10.1038/nature16152 (2015).

21 Anthony, D. W. *et al.* The Samara valley project. *Late Bronze Age economy and ritual in the Russian steppes. Eurasia Antiq* **11**, 395-417 (2005).

22 Peltzer, A. *et al.* EAGER: efficient ancient genome reconstruction. *Genome Biol* **17**, 60, doi:10.1186/s13059-016-0918-z (2016).

23 Li, H. & Durbin, R. Fast and accurate long-read alignment with Burrows–Wheeler transform. *Bioinformatics* **26**, 589-595 (2010).

24 Renaud, G., Stenzel, U. & Kelso, J. leeHom: adaptor trimming and merging for Illumina sequencing reads. *Nucleic Acids Res.*, e141 (2014).
25 Skoglund, P., Storå, J., Götherström, A. & Jakobsson, M. Accurate sex identification of ancient human remains using DNA shotgun sequencing. *J. Archaeol. Sci* **40**, 4477-4482 (2013).
26 Mittnik, A., Wang, C.-C., Svoboda, J. & Krause, J. A Molecular Approach to the Sexing of the Triple Burial at the Upper Paleolithic Site of Dolní Věstonice. *PloS One* **11**, e0163019 (2016).
27 Rasmussen, M. *et al.* An Aboriginal Australian genome reveals separate human dispersals into Asia. *Science* **334**, 94-98 (2011).
28 Korneliussen, T. S., Albrechtsen, A. & Nielsen, R. ANGSD: analysis of next generation sequencing data. *BMC Bioinformatics* **15**, 356 (2014).
29 Peltzer, A. *et al.* EAGER: efficient ancient genome reconstruction. *Genome Biol* **17**, 60, doi:10.1186/s13059-016-0918-z (2016).
30 Renaud, G., Slon, V., Duggan, A. T. & Kelso, J. Schmutzi: estimation of contamination and endogenous mitochondrial consensus calling for ancient DNA. *Genome Biol* **16**, 224, doi:10.1186/s13059-015-0776-0 (2015).
31 Fan, L. & Yao, Y.-G. An update to MitoTool: using a new scoring system for faster mtDNA haplogroup determination. *Mitochondrion* **13**, 360-363 (2013).
32 Lazaridis, I. *et al.* Genomic insights into the origin of farming in the ancient Near East. *Nature* **536**, 419-424 (2016).
33 Mittnik, A. *et al.* The genetic prehistory of the Baltic Sea region. *Nat Commun* **9**, 442, doi:10.1038/s41467-018-02825-9 (2018).
34 Fu, Q. *et al.* The genetic history of Ice Age Europe. *Nature* **534**, 200-205, doi:10.1038/nature17993 (2016).
35 Jones, E. R. *et al.* The Neolithic transition in the Baltic was not driven by admixture with early European farmers. *Curr. Biol.* **27**, 576-582 (2017).
36 Patterson, N., Price, A. L. & Reich, D. Population structure and eigenanalysis. *PLoS Genet.* **2**, e190 (2006).
37 Price, A. L. *et al.* Principal components analysis corrects for stratification in genome-wide association studies. *Nat. Genet.* **38**, 904-909 (2006).
38 Price, A. L., Zaitlen, N. A., Reich, D. & Patterson, N. New approaches to population stratification in genome-wide association studies. *Nat. Rev. Genet.* **11**, 459-463 (2010).
39 Alexander, D. H., Novembre, J. & Lange, K. Fast model-based estimation of ancestry in unrelated individuals. *Genome Res.* **19**, 1655-1664 (2009).
40 Purcell, S. *et al.* PLINK: a tool set for whole-genome association and population-based linkage analyses. *Am. J. Hum. Genet.* **81**, 559-575 (2007).
