# Supplementary material for: Analysis of 3800-year-old Yersinia pestis genomes suggests Bronze Age origin for bubonic plague
Source: Nat Commun. 2018 Jun 8;9:2234. doi: 10.1038/s41467-018-04550-9 (PMC5993720; doi:10.1038/s41467-018-04550-9)
Supplement: Supplementary file 3 — Description of Additional Supplementary Files [file 41467_2018_4550_MOESM3_ESM.pdf]

Description of Additional Supplementary Files

File Name: Supplementary Data 1
Description: SNP table depicting all positions variant in RT5.

File Name: Supplementary Data 2
Description: Table of *Y. pestis* genomes used in this study for phylogenetic analysis

File Name: Supplementary Data 3
Description: *Y. pestis* SNP positions leading to RT5, and their respective alleles in RISE397.

File Name: Supplementary Data 4
Description: *Y. pestis* SNP positions between RT5 and Justinian2148, and their respective alleles in RISE397.
